# Evidence for an Association Between Hearing Impairment and Disrupted Sleep: Scoping Review

**DOI:** 10.1044/2019_AJA-19-0026

**Published:** 2019-10-17

**Authors:** Nathan A. Clarke, Derek J. Hoare, Edward C. Killan

**Affiliations:** a National Institute of Health Research Nottingham Biomedical Research Centre, of Nottingham, United Kingdom; b Hearing Sciences, Division of Clinical Neuroscience, School of Medicine, University of Nottingham, Nottingham, United Kingdom; c Audiological Science and Education Group, Leeds Institute of Cardiovascular and Metabolic Medicine, Faculty of Medicine and Health, University of Leeds, United Kingdom

## Abstract

**Purpose:**

Hearing impairment (HI) is the most common sensory impairment and may negatively impact sleep through reduced auditory input. Factors associated with HI such as anxiety regarding communication in daily life may also adversely impact an individual's sleep. Here, research on the relationship between HI and sleep disruption was catalogued using scoping review methodology.

**Method:**

A systematic strategy was employed to search various electronic databases. This review is reported according to the Preferred Reporting Items for Systematic Review and Meta-Analyses Scoping Review Extension.

**Results:**

Sixteen records met inclusion criteria. Studies have investigated sleep in HI as a primary aim in noise-exposed workers or large surveys in older participants. Experimental and quasi-experimental studies report alterations to sleep architecture of potential neuroplastic origins. Studies reporting sleep as a secondary aim generally report poorer sleep in HI participants.

**Conclusions:**

This scoping review has catalogued evidence that altered or negatively impacted sleep may be associated with HI. Potential confounding factors, mechanisms, and considerations for future research are discussed.

**Supplemental Material:**

https://doi.org/10.23641/asha.9968369

All sensory processing continues during sleep, though hearing has been uniquely described as the “sentinel sense” for its role in continuously monitoring our surroundings (Velluti, 2018). It is intuitive that the organization of sleep is extremely sensitive to acoustic stimuli; one needs only to have awoken to a loud sound at night to have reached this conclusion. However, a complex, reciprocal relationship exists between sleep and the auditory system (Velluti & Pedemonte, 2012). The absence of acoustic stimuli has also been shown to alter sleep (Velluti, 2018). Hearing impairment (HI) is a permanent reduction in unaided hearing thresholds and is the most common sensory impairment, representing a significant global disease burden (Correia et al., 2016; Pascolini & Smith, 2009). Based on animal studies, reduction to an individual's auditory input through HI has the potential to adversely impact sleep, however, whether a causal mechanism exists in humans between sleep disruption and reduced auditory input per se remains an open question.

Knowledge of the potential for acoustic deprivation to alter sleep is not recent. Postulation of a “continuous sleep,” lasting “99% of the time,” was made following surgical section of olfactory, optic, acoustic, and trigeminal nerves in cats (Hagamen, 1959; Velluti, 2018). However, further work in this model using polysomnography revealed a sleep–wake cycle of altered characteristics; these included a reduction in total sleep time and reductions to the relative distribution of sleep stages (i.e., sleep architecture), with less slow-wave sleep (SWS) and rapid eye movement (REM) stages (Vital-Durand, 1971). Other animal models have demonstrated that diminished auditory input causes alterations to sleep. Pedemonte, Peña, Torterolo, and Velluti (1997) found that bilateral surgical removal of the cochleae of guinea pigs changed their sleep architecture, with enhancement of both SWS and REM stages. The authors also noted decreased wakefulness accompanying these changes. Later, Cutrera et al. (2000) demonstrated that cochlear lesions caused alterations to circadian and ultradian rhythms in golden hamsters. Although the results of these animal studies are consistent with a view that HI negatively impacts sleep, comparisons between human and animal sleep should be made cautiously due to diurnality and nocturnality in different species. Furthermore, cochlear destruction and auditory nerve section may not be optimal models for representing a causal relationship between reduced auditory input through HI and altered/disrupted sleep because they entail sudden, complete, and traumatic removal of auditory input.

The “auditory deprivation” mechanism of disrupted sleep warrants further investigation alone; however, other plausible mechanisms of HI disrupting sleep exist. For instance, anxiety is commonly caused by HI and may therefore impact sleep via a “communication anxiety” mechanism (Monzani, Galeazzi, Genovese, Marrara, & Martini, 2008; Staner, 2003). Furthermore, the range of HI that can be experienced in humans (i.e., mild–profound deafness) necessitates a further investigation of the association between sleep and HI.

The importance of sleep for an individual's overall health has become well understood in recent years. Enhanced sleep has been linked to improved cognitive outcomes such as enhanced memory consolidation (Ngo, Martinetz, Born, & Mölle, 2013; Ong et al., 2016), whereas insufficient sleep is associated with poorer physical health outcomes (Kecklund & Axelsson, 2016). Disrupted and poor-quality sleep is associated with numerous deleterious health outcomes, including cardiovascular and metabolic diseases (Diekelmann & Born, 2010; Luyster et al., 2012). Disrupted sleep through HI may therefore indirectly contribute to worsening of cognitive performance and disease outcomes in those with HI.

Understanding the nature of the relationship between HI and sleep disruption is crucial, due to both the prevalence of HI and the wider impact of disrupted sleep on health. Although animal evidence is suggestive of HI altering sleep, a summary of the available evidence of the impact of HI on human sleep is required. As no review has previously investigated the relationship between HI and sleep disruption, it is appropriate to catalogue current knowledge on this topic in a scoping review.

Scoping reviews are designed to rapidly map the “key concepts underpinning a research area and the main sources and types of evidence available.” (Fulop, 2001, p. 194). Munn et al. (2018) note that use of scoping review methodology is indicated

- to identify the types of evidence available in a given field,
- to clarify key concepts/definitions within the literature,
- to examine how research is conducted on a certain topic or field,
- to identify key characteristics or factors related to a concept,
- as a precursor to a systematic review, and
- to identify and analyze knowledge gaps.

The primary aim of this review was to scope and catalogue research addressing the proposed association between HI and disrupted sleep in humans. Potential confounding factors, mechanisms, and considerations for future research concerning HI and disrupted sleep are also discussed.

## Method

This review is reported according to the Preferred Reporting Items for Systematic Review and Meta-Analyses Extension for Scoping Reviews (Tricco et al., 2018). It was conducted in five stages according to the methodology described in Arksey and O'Malley (2005).

### 1. Identifying the Research Question

This review aimed to answer the question: Is there evidence for an association between HI and sleep difficulties, and does it (a) facilitate an understanding of the scope and types of evidence that exist within the peer-reviewed literature; (b) catalogue the extent, range, and nature of research activity on this topic; and (c) identify gaps in the research literature.

### 2. Identifying Relevant Studies

#### Eligibility Criteria

Records were included if they reported a sleep-related outcome measure in human participants who have HI. Because of the established differences in sleep between adults and children, only studies that included adult participants (≥ 18 years old) were considered within this review (Edwards et al., 2010). Review articles were excluded. Only studies published within a peer-reviewed journal and written in English (or with an existing English translation available) were included. Studies primarily investigating the established association between tinnitus and disrupted sleep were excluded. Studies investigating the impact of sleep apnea on HI were also excluded. No date restriction was placed on retrieval of records.

#### Search Strategy

To identify relevant studies, a search strategy was discussed and developed among authors with relevant terms and databases selected. The selected databases were PubMed, PsychINFO (via Ovid SP), CINAHL (via EBSCO Host), and Web of Science (Science and Social Science Citation Index). These databases adequately encompass the wide range of multidisciplinary interests pertaining to the areas of sleep and hearing research in human participants. The search strategy utilized a combination of the following Medical Subject Headings terms and keywords to create the following search string, which was adapted to search each database:

((hearing loss OR hearing impair* OR deaf*) AND (sleep OR insomnia))

Initial electronic databases searches were conducted in September 2016. Further hand searches for relevant records were conducted by screening the reference lists of included studies and screening the table of contents of key journals and Google Scholar for records published since the initial searches. Update searches were undertaken in August 2018.

### 3. Study Selection

Search results were independently examined by two researchers. Results were initially screened by title and abstract, with irrelevant studies being discarded. Potentially relevant studies or studies where the title and abstract provided insufficient screening information to make a judgment were then screened by full text. Disagreements regarding the inclusion of a study within the review were resolved through discussion between the authors or the third member of the review team being consulted to adjudicate. A total of 16 records were included in the final review.

### 4. Charting the Data

A data-charting form was developed by the authors and piloted using three records. Authors then refined the included items via iterative discussion to best reflect the aims of the review. Two researchers extracted data independently across all records. Data items included study identification, authors and year of publication, country of study origin, number of participants in sample, age of sample participants, intervention used in study, study design, HI measurements used, HI of participants, management of HI, sleep measurements used, reported sleep of participants, other outcome measures used, presence and severity of tinnitus, main findings, and future research suggestions. Independently extracted datasets were compared, and any disagreements in what was extracted were resolved through discussion between authors until consensus was achieved on a single extracted data form (see Supplemental Material S1).

### 5. Collating, Summarizing, and Reporting the Results

Evidence was characterized according to the key themes by which they were charted: aim of study, sample size, study design, HI measurements, reported level sample HI, measurements, sleep of participants, presence and severity of tinnitus, and future research suggestions. It is not applicable to assess the risk of bias (i.e., make a judgment about relative study quality) within scoping review methodology (Tricco et al., 2018), though initial critical appraisal of studies was determined by important factors such as by potential confounds and measurement of participant hearing. It is already known that tinnitus is associated with disturbed sleep, and tinnitus is often found with HI. It was therefore relevant to make an initial appraisal of whether the included records had considered this important variable. Measurement of HI and the study samples was also critically appraised to better understand the known relationship between sleep and the wide range of HI that is possible in humans. Finally, we hypothesized a mechanism by which anxiety may lead to disrupted sleep for individuals with HI, particularly those of an older working age (i.e., where the effects of age-related HI may be manifest and contributing to communication difficulties, but amplification or rehabilitation has not been provided). We therefore also consider evidence that highlights mechanisms by which HI may disrupt an individual's sleep. Finally, to facilitate the identification of gaps in the research literature, we considered the nature of the catalogued evidence and how future work on this question could develop.

## Results

**Figure 1.**
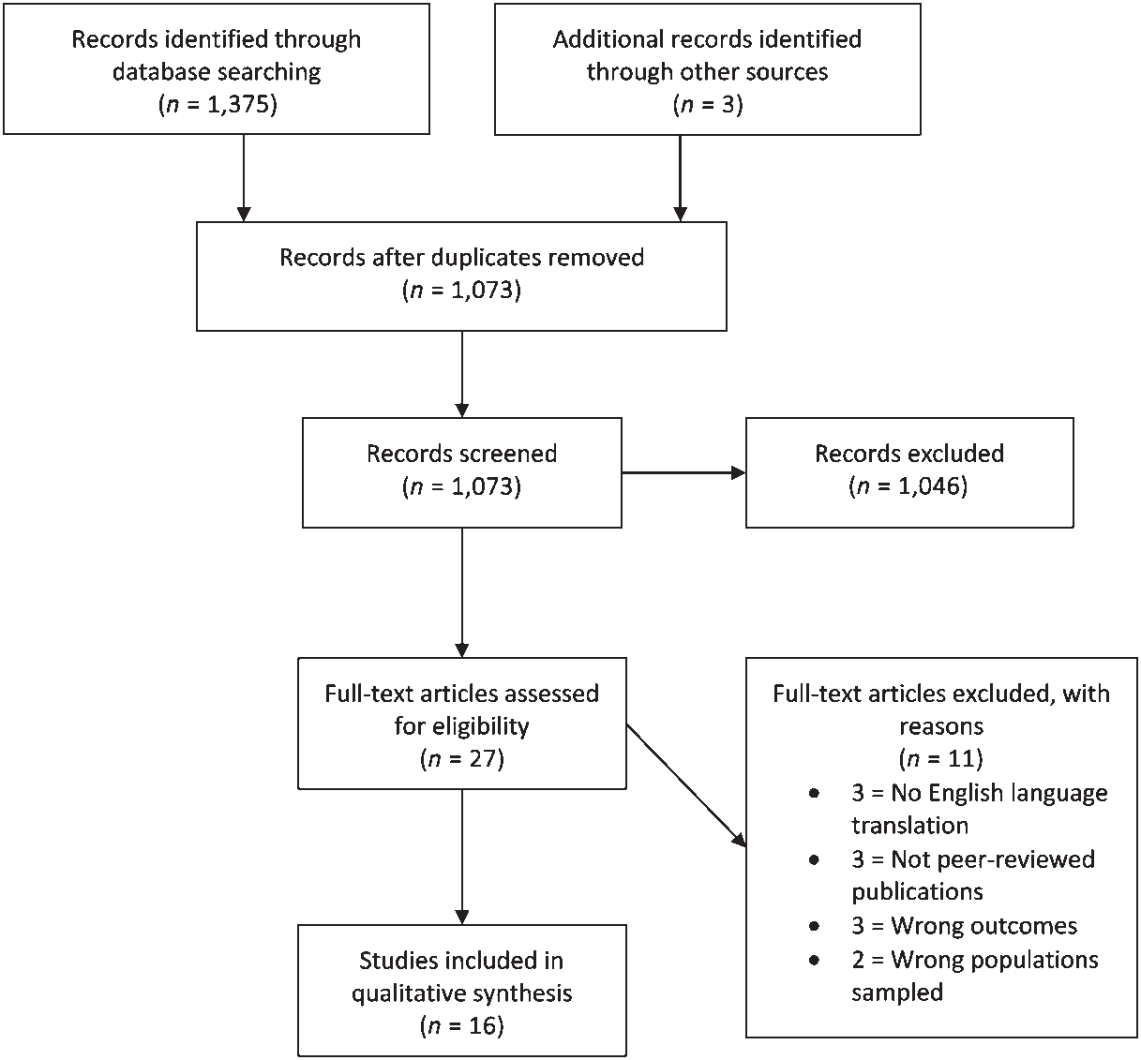
Flow diagram detailing the study search and record selection process.

Sixteen records met the inclusion criteria for this review. Figure 1 details a flowchart of the study search and record selection process.

### Evidence for an Association Between HI and Disrupted Sleep

#### Studies Containing Sleep Outcomes as a Primary Aim

Nine studies reported a primary aim of investigating the association between HI and/or sleep quality or structure, with two studies having directly investigated sleep in participants with HI related to noise exposure. The first of these, Test, Canfi, Eyal, Shoam-Vardi, and Sheiner (2011) aimed to investigate sleep quality in participants who had HI following prolonged occupational noise exposure. Pure-tone audiometry (PTA) was performed at 250–8000 Hz, with HI defined as average threshold > 25 dB HL at 1–4 kHz. A sample of 298 participants from a working-age population was included, comparing individuals with HI and individuals without. This study used the Mini Sleep Questionnaire (Zomer, Peled, Rubin, & Lavie, 1985) and reported higher median scores for the HI group compared to controls. Subgroup analyses of sleep quality data were undertaken with multivariate linear regression and logistic regression models, grouped via participant age (younger or older than 50 years old) and presence of tinnitus (established by a single yes or no question). The authors reported that HI was “inversely associated with sleep quality, while tinnitus was the main sleep disrupting factor.” Similarly, Lim et al. (2017) aimed to investigate the association between HI and insomnia in a sample of 809 noise-exposed male workers. They reported that PTA thresholds at 1–2 kHz in the right ear and 1 kHz were considerably higher among workers with insomnia. The authors calculated an odds ratio of 1.806 for insomnia occurring with HI (confidence interval = 1.022–3.188, *p* = .042) after adjusting for other variables.

A number of studies have evaluated large samples of participants through surveys or medical databases. Asplund (2003) aimed to investigate the relationship between hearing complaints, sleep, and daytime sleepiness in a sample of 6,143 elderly participants with self-reported HI. The study reported an increased incidence of daytime sleepiness in HI participants independent of health quality status. The author also noted that participants with HI reported poorer sleep, more frequent awakenings, and difficulty returning to sleep if awoken. Nakajima, Kanda, Hosobuchi, and Suwa (2014) performed a large study with both cross-sectional and longitudinal arms, aiming to discover whether subclinical HI is associated with sleep duration and cardiometabolic risk factors. The authors report that HI is independently associated with longer sleep duration in a sample of 6,774 participants in all age groups (except 20–39-year-olds). In a follow-up study, Nakajima, Kanda, and Suwa (2018) aimed to investigate the association between subclinical hearing loss and self-reported restorative sleep (determined by a positive response to the question “Do you get sufficient rest from sleep?”), in a database of middle-aged and elderly Japanese people. They reported that bilateral subclinical hearing loss (defined as thresholds > 25 dB at 4000 Hz) was statistically significantly associated with restorative sleep, and this effect was independent of age, sex, and cardiometabolic risk factors.

Other studies have looked into sleep-related outcomes in HI through experimental or quasi-experimental designs. Velluti, Pedemonte, Suárez, Bentancor, and Rodríguez-Servetti (2010) report that sleep organization is altered within a sample of profoundly deaf participants with cochlear implants. The study used electroencephalography (EEG) to investigate whether auditory input alters sleep structure in a sample of nine participants, four of whom had previously received cochlear implants. Participant's overall sleep structure was found to be no different when compared with normal-hearing individuals. However, statistically significant within-group increases in EEG activity were reported for Stage 2 and REM sleep for the implanted participants when the device was turned off; the authors attributed this to neural plasticity induced by a lack of auditory input. Scrofani et al. (1996) used EEG to investigate sleep spindles (SS; a distinctive, non-REM, thalamocortical EEG oscillation) activity in four deaf participants, as part of a larger study into pathological deafferentation of somatosensory and sensory inputs. The authors report statistically significant increases in SS activity, including increased SS frequency and duration in deaf participants compared with controls. Nakayama et al. (2010) aimed to evaluate sleep quality using polysomnography in a sample of 35 participants with refractory Ménière's disease. Total sleep duration was significantly longer in duration compared to controls. The authors also reported that Stage 2 sleep was increased in participants with Ménière's, whereas Sleep Stages 3 and 4 were reported to be decreased. Arousal from sleep was also increased compared to control participants. Zarenoe, Hallgren, Andersson, and Ledin (2017) aimed to investigate sleep, working memory, and hearing problems in a group of patients with sensorineural hearing loss (SNHL) and tinnitus, compared to a control group of participants with SNHL only, before and after hearing rehabilitation. They reported a statistically significantly poorer score at baseline using the Pittsburgh Sleep Quality Index (PSQI; Buysse, Reynolds, Monk, Berman, & Kupfer, 1989) for participants with SNHL and tinnitus compared with SNHL only (in both a full and age-matched analysis). There was a statistically significant reduction in sleep scores following intervention with hearing aids, indicating sleep improvement after rehab in the SNHL and tinnitus group but not SNHL alone.

#### Studies Containing Sleep Outcomes as a Secondary Aim

Seven studies did not have the primary aim of investigating the impact of HI on sleep but reported sleep-related outcomes. In general, these studies suggested alterations or poorer sleep in HI participants compared to those without HI. Jansson-Fröjmark et al. (2012) aimed to investigate the effect of cognitive behavioral therapy for insomnia on patients with insomnia and comorbid HI, reporting that poor sleep quality was associated with hearing problems. Hasson, Theorell, Wallén, Leineweber, and Canlon (2011) investigated the prevalence of hearing complaints and tinnitus in relation to various work-related stressors. They reported a statistically significant difference in the prevalence of hearing problems (defined as having hearing complaints, tinnitus, or both) in participants with different levels of sleep quality (as assessed by a single 5-point scale item asking, “How is your sleep quality in general?”). Participant reports of either hearing problems, tinnitus, or both were found to increase as a function of poorer sleep quality. Tomioka, Okamoto, Morikawa, and Kurumatani (2015) investigated the relationship between self-reported HI and 5-year cognitive decline in high-functioning elderly adults in a longitudinal study. They reported a statistically significant difference between HI and normal-hearing groups, as measured by the PSQI. At baseline, 37.6% of HI participants reported a score greater than 5.5 (indicating poorer sleep), compared to 31.9% of those with normal hearing. Results were based on self-report of 3,936 elderly participants' perception of whether they had HI.

A number of studies investigated health-related quality of life in participants with HI, which included various sleep-related outcomes. Dalton et al. (2003) aimed to investigate the impact of HI on quality of life in older adults. They reported statistically significant differences between groups in a sample of 2,688 adults with mild, moderate to severe, and no HI for the item “sleep problems” on the Short Form 36 Health Survey. Werngren-Elgström, Dehlin, and Iwarsson (2003) investigated health-related quality of life (expressed as subjective well-being and perceived symptoms) among prelingually deafened elderly adults who used sign language. They reported markedly higher prevalence of insomnia among this population, and interruptions to their sleep, with nearly half struggling to return to sleep once awoken. Ringdahl and Grimby (2000) aimed to measure health-related quality of life in severe-to-profound postlingually deafened adults. They report severe-to-profound HI (defined as SNHL in the better ear of 70 dB HL at 1 kHz) as having a significant impact on participants' “energy resources.” Danermark and Gellerstedt (2004) aimed to describe the health status of HI individuals' working life in terms of psychosocial well-being and discuss these in relation to a demand-control model. HI individuals with high-stress jobs reported more health problems than other groups in study (grouped by demand-control theory into high stress, low stress, and active and passive). This included reporting the most sleep problems of any group.

In summary, various studies have reported preliminary evidence for an association between HI and disrupted sleep. Nine have investigated sleep in HI participants as a primary aim, with studies having used a variety of designs, and assessed a wide age range of participants and sample sizes. Relevant key findings include a reported association between HI and sleep that is independent of tinnitus, reported alterations in sleep structure in HI participants, and suggestion of neuroplastic mechanisms for HI disrupting sleep. Seven studies have incorporated sleep-related outcomes in HI samples as a secondary aim, with many primarily interested in health-related quality of life. Findings from these studies suggest that sleep is poorer in HI individuals, with poorer scores reported in sleep and quality of life measures.

### HI Populations and Their Measurement

Populations with HI ranging from mild HI to profound deafness were sampled within the included records. Two records included samples with prelingually deafened participants, with only one record stating its participants as being congenitally deafened (Scrofani et al., 1996; Werngren-Elgström et al., 2003). Approximately half of the records obtained information about the sampled participants' hearing thresholds, whereas the others relied on self-report. Werngren-Elgström et al. (2003) did not include any measure of HI, as their study sampled profoundly, prelingually deafened individuals. Similarly, Nakayama et al. (2010) did not report a hearing measurement in their study, although HI was assumed as they sampled patients with a confirmed diagnosis of Ménière's disease (which typically features a unilateral, fluctuating HI). Scrofani et al. (1996) included participants that were described as having been deafened from birth by rubella. Deafness was confirmed using brainstem auditory evoked potentials. Hasson et al. (2011) obtained their results from a sample of 9,756 participants who were defined as having hearing complaint if they reported finding conversation in a group setting “difficult” or “quite difficult.” Velluti et al. (2010) reported using speech audiometry to verify hearing status of participants of their study that sampled patients with cochlear implants. Tomioka et al. (2015) asked their participants to answer the questions “Do you feel you have a hearing loss?”, with three response options (“yes,” “no,” or “I don't know”). Asplund (2003) asked their sampled participants to confirm or deny the binary statement of “I have good hearing.”

Nine studies used PTA to measure hearing across 250–8000 Hz. Nakajima et al. (2018) reported using PTA for hearing threshold measurement but did not report the total number of frequencies assessed in their sample. The variance in PTA criteria (if measured at all) currently makes direct comparison of the sleep-related results obtained across the studies challenging and impractical. Verification of hearing thresholds in participants using PTA is crucial as it would allow for comparison between samples when synthesizing the available literature with systematic review and meta-analysis. Furthermore, hearing handicap (i.e., the perceived functional impact of HI) is separable from hearing thresholds. For example, someone with only a “moderate” HI when averaged across measured PTA frequencies may experience a severe level of handicap, increasing their distress. It is therefore an important variable to consider in any study investigating a relationship between HI and disrupted sleep.

### Tinnitus as a Confounding Factor

Tinnitus has an established negative association with sleep (Wallhäusser-Franke, Schredl, & Delb, 2013). It is a problematic confound when assessing the association between sleep and HI, as tinnitus is rarely present without some degree of HI (Rauschecker, Leaver, & Mühlau, 2010). An additional complicating factor is that both HI and tinnitus incidence increase with age (Martinez, Wallenhorst, McFerran, & Hall, 2015). Nine studies in this review did not report assessment of tinnitus status of participants in their samples (Asplund, 2003; Dalton et al., 2003; Lim et al., 2017; Nakajima et al., 2018; Ringdahl & Grimby, 2000; Scrofani et al., 1996; Tomioka et al., 2015; Velluti et al., 2010; Werngren-Elgström et al., 2003). Nakayama et al. (2010) do not explicitly mention tinnitus, but it is assumed to be included as part of the definite Ménière's disease diagnosis criterion. Only one study measured the severity of tinnitus using an established tinnitus questionnaire (Zarenoe et al., 2017).

For records that reported tinnitus status of participants, the definition and inclusion criteria varied significantly. Individuals reporting bothersome tinnitus in the absence of HI may, in fact, have an unidentified HI (according to typical clinical standards) unknowingly contributing to sleep disruption. Conversely, many tinnitus sufferers are not sufficiently disturbed by the percept to report it, making it difficult to attribute disrupted sleep to HI per se.

In summary, tinnitus appears poorly considered within the available literature concerning the association between HI and disrupted sleep. The lack of consistency in reporting will make synthesis of the current literature on this topic problematic. The need for improved reporting of outcome measures for studies involving tinnitus has been gaining increased attention from researchers (Hall et al., 2016). Due to the known disruptive impact of tinnitus on sleep, future studies investigating the impact of HI on sleep should consider measuring this important confound.

## Discussion

To the authors' knowledge, this is the first review to investigate evidence concerning negative impacts or alterations to sleep associated with HI. The review catalogues currently available evidence for an association between HI and disrupted sleep in humans. However, whether auditory deprivation per se alters or disrupts sleep remains an open question. Important confounds could account for the catalogued association such as presence/severity of tinnitus and adequate measurement of hearing thresholds. Furthermore, if an association between disrupted sleep and HI exists in the absence of such confounds, it may do so through moderating/mediating mechanisms that are discussed below. The review has also demonstrated a paucity of studies with the primary aim of investigating the association between HI and altered or disrupted sleep featuring heterogeneous samples. Moreover, synthesis of evidence regarding the proposed association will be challenging due to heterogeneity of measures used to assess sleep quality and additional heterogeneity of HI participant samples. Heterogeneity of HI participants is an important issue, as both severity of HI (e.g., mild–profound) and onset (e.g., congenital or acquired) could have implications for any associative mechanism between disrupted sleep and HI. Here, we consider mechanisms that could be responsible for an association between HI and disrupted sleep. These include neuroplastic changes triggered by diminished auditory input, or altered/disrupted sleep or HI being the consequence of an unknown third variable.

## HI Disrupting Sleep: Potential Mechanisms

Sleep alternates between REM and non-REM cycles, with non-REM sleep having “light” and “deep” sleep distinctions. Deep sleep or SWS is split into four stages (I–IV), accompanied by physiological markers including reduced heart rate and blood pressure. Sleep spindles mark the onset of Stage II sleep and have been implicated in preventing arousal through inhibiting information processing (Roebuck et al., 2014).

### Auditory Deprivation

Evidence in this review suggests that HI is associated with specific alterations to sleep architecture (i.e., the cyclic structure of sleep throughout the night). Reported alterations include increased overall sleep duration and altered EEG measures of sleep architecture compared to controls, including alterations to the amount of time spent in various sleep stages, and increased SS frequency and duration (Nakajima et al., 2014; Nakayama et al., 2010; Scrofani et al., 1996; Velluti et al., 2010). Reports regarding which specific sleep stages are associated with HI are mixed. Velluti et al. (2010) report increases to Stage II and REM sleep, whereas Nakayama et al. (2010) report an increase in Stage II and a decrease in Stages III and IV. Poorer subjective sleep quality is also reported through increased measures of frequency of arousal, as well as reported difficulties returning to sleep and reduced energy for HI individuals (Nakayama et al., 2010; Ringdahl & Grimby, 2000; Werngren-Elgström et al., 2003).

Neuroplastic alterations to sleep were suggested by Velluti et al. (2010) as a consequence of changes to auditory input. The authors reported a statistically significant difference in sleeping EEG results in cochlear implant users between device “on” and “off” conditions. This study provides quasi-experimental evidence for reduced auditory input altering sleep structure. Previous animal research has demonstrated reduced wakefulness and increased durations of REM sleep following total removal of auditory stimulus in animals (Cutrera et al., 2000; Pedemonte et al., 1997). Velluti et al. (2010) also report increases in amounts of REM sleep in participants deprived of night-time sound. Taken together, these results suggest neural plasticity through diminished auditory input may result in negative alterations to sleep patterns. If true, this would raise clinical questions, such as whether providing amplification at night could help to mitigate negatively impacted sleep in patients with HI through providing access to ambient sound.

### Communication Anxiety

We suggest that HI could also adversely impact sleep by causing or increasing anxiety, a negative psychological state that is already known to negatively impact sleep (Danermark & Gellerstedt, 2004; Staner, 2003). Anxiety may be increased through anticipation or experience of communication difficulties in challenging work or social environments. HI may also result in increased anxiety and reduced psychological well-being. Thus, there is potential for HI to alter or negatively impact sleep through anxiety.

Anxiety and its effect on sleep may be worsened or potentially caused by HI. Anxiety and stress have also been associated with individuals who overestimate the severity of their HI (Kim et al., 2017). Moreover, mild–moderate HI has been shown to negatively impact sociosituational functioning and increase anxiety in middle-aged adults (Monzani et al., 2008). Anxiety is likely to be worse when combining HI and a high-stress job type, so it could be particularly prevalent in a modern, middle-aged, working population (Danermark & Gellerstedt, 2004). Indeed, mental health ratings for those under 65 years old with HI have been reported to be negatively impacted, with HI substantially increasing scores on measures of depression and loss of self-esteem (Tambs, 2004). Monzani et al. (2008) suggest that anxiety and its impact on psychological well-being may be due to a fear of communicating in acoustically challenging situations and environments, which are likely a constant consideration for HI adults when working or socializing. Although those of working age may be more likely to experience disrupted sleep through anxiety related to HI, associations have also been reported between HI and disrupted sleep in the elderly (Asplund, 2005; Jansson-Fröjmark et al., 2012). This review identified tentative support for the communication anxiety hypothesis through evidence that HI workers with high-stress jobs reported more sleep problems than workers with less stressful roles, suggesting an interaction between these variables (Danermark & Gellerstedt, 2004).

### Other Mechanisms

A further possibility is that a third variable such as Alzheimer's or cardiac disease (Nakajima et al., 2014) mediates the association between HI and disrupted sleep. A person's peripheral hearing thresholds (as measured by PTA) may indicate impaired central nervous and/or cardiovascular function that results in poorer sleep. A similar “common cause” possibility has already been suggested with regard to the reported association between HI and cognitive decline (Dawes et al., 2015). Although sleep is becoming increasingly understood as essential to healthy functioning, the link between HI and sleep is only beginning to be elucidated, with new animal research suggestive of sleep deprivation having a reciprocally deleterious effect on hearing (Jung et al., 2018). If the conclusions of such research are applicable to humans, then a negative feedback mechanism may exist between sleep and hearing where HI disrupts sleep, which, in turn, worsens hearing thresholds.

Based on current catalogued evidence, it is difficult to make specific clinical recommendations. Assuming an association between sleep and HI, the detection and intervention of HI become increasingly important for clinicians as the wider health burden of disrupted sleep through HI could be ameliorated with an appropriate hearing intervention (Diekelmann & Born, 2010; Luyster et al., 2012). Such proactive interventions promote increased understanding of a model of health care delivery that acknowledges the complex relationships that exist in effective holistic patient management. Furthermore, this review highlights the importance of adequate measurement. HI patients presenting with disrupted sleep should, therefore, have specific aspects of their sleep disruption scrutinized (e.g., sleep latency issues, frequent awakenings, daytime lethargy). Clinicians may wish to administer established instruments to formally assess disrupted sleep, such as the PSQI. Clinicians may then wish to consider referring their patient to a sleep medicine specialist if deemed appropriate (Buysse et al., 1989).

## Review Limitations and Future Research

Depression and advanced age are both known to have significant negative associations with the quality of an individual's sleep (Edwards et al., 2010; Murphy & Peterson, 2015). Elderly individuals have been reported to be less negatively impacted by HI than younger and middle-age individuals (Tambs, 2004). Diminished negative impact may include reduced sleep disruption caused by HI. Therefore, future studies should also investigate sleep in younger and middle-age samples, who are likely to have more social and work commitments and may be more functionally handicapped by HI. Although several studies in this review did measure depression as part of investigations into quality of life, most did not. As depression is known to alter sleep, it should be considered in future research concerning the association between HI and sleep.

Limitations of this review should be duly considered. This includes searches being limited to electronic databases and peer-reviewed publications. As scoping reviews are an effort to gather information from a wide variety of sources, reports from non–peer-reviewed sources such as gray literature and searching of hard copy journals may also be considered as preliminary evidence for the association between HI and sleep disruption. However, the potential quality of non–peer-reviewed literature should also be considered.

Scoping review methodology only aims to map the nature of the evidence available on a specific topic; it does not comprehensively formally appraise the quality of evidence (Tricco et al., 2018). Although the included studies suggest that HI is associated with poorer sleep outcomes, there are several confounds in the evidence. This requires that interpretations be made cautiously in the context of future research. Obvious potential confounds that may hamper future research include sampled participant's age, severity of HI, and tinnitus status. Due to the prevalence of these factors in the literature, each should be addressed and measured in future studies to improve understanding of the nature of the relationship between HI and reduced or altered sleep. Improved verification of HI in the sampled participants should be addressed going forward. There were a variety of verification methods used in those studies that included an objective measurement of hearing thresholds, including PTA, speech audiometry, and auditory evoked potentials. However, many studies relied on participants self-reporting whether or not they had HI. This is problematic as there are potential reliability issues regarding the accuracy of self-reported hearing handicap compared with audiometric measurements (Brainerd & Frankel, 1985). Future studies investigating the relationship between HI and sleep should therefore include PTA verification of HI in sampled participants. This will facilitate comparisons across studies, and enable investigation of pertinent questions which follow on from preliminary research, such as whether severity of HI plays a role in sleep disturbance.

## Conclusion

This scoping review has catalogued evidence that altered or negatively impacted sleep may be associated with HI. As poor quality or interrupted sleep has numerous negative effects on health, it is important to further understand the nature of the relationship between sleep and HI. Potential mechanisms for this association include auditory deprivation and communication anxiety. Catalogued evidence is inconclusive as to the relation of HI severity, or onset, to these potential mechanisms. Highlighted factors such as tinnitus, age, and depression should be routinely considered as confounds when investigating the relationship between HI and disrupted sleep. Future research should also focus on providing evidence that is able to differentiate outlined theoretical mechanisms to enable an increased understanding of the catalogued association between HI and disrupted sleep.

## Supplementary Material

10.1044/2019_AJA-19-0026SMS1 Supplemental Material 1. Table of extracted data for included records.
